# Identifying and Seeing beyond Multiple Sequence Alignment Errors Using Intra-Molecular Protein Covariation

**DOI:** 10.1371/journal.pone.0011082

**Published:** 2010-06-28

**Authors:** Russell J. Dickson, Lindi M. Wahl, Andrew D. Fernandes, Gregory B. Gloor

**Affiliations:** 1 Department of Biochemistry, The University of Western Ontario, London, Canada; 2 Department of Applied Mathematics, The University of Western Ontario, London, Canada; Institute of Infectious Disease and Molecular Medicine, South Africa

## Abstract

**Background:**

There is currently no way to verify the quality of a multiple sequence alignment that is independent of the assumptions used to build it. Sequence alignments are typically evaluated by a number of established criteria: sequence conservation, the number of aligned residues, the frequency of gaps, and the probable correct gap placement. Covariation analysis is used to find putatively important residue pairs in a sequence alignment. Different alignments of the same protein family give different results demonstrating that covariation depends on the quality of the sequence alignment. We thus hypothesized that current criteria are insufficient to build alignments for use with covariation analyses.

**Methodology/Principal Findings:**

We show that current criteria are insufficient to build alignments for use with covariation analyses as systematic sequence alignment errors are present even in hand-curated structure-based alignment datasets like those from the Conserved Domain Database. We show that current non-parametric covariation statistics are sensitive to sequence misalignments and that this sensitivity can be used to identify systematic alignment errors. We demonstrate that removing alignment errors due to 1) improper structure alignment, 2) the presence of paralogous sequences, and 3) partial or otherwise erroneous sequences, improves contact prediction by covariation analysis. Finally we describe two non-parametric covariation statistics that are less sensitive to sequence alignment errors than those described previously in the literature.

**Conclusions/Significance:**

Protein alignments with errors lead to false positive and false negative conclusions (incorrect assignment of covariation and conservation, respectively). Covariation analysis can provide a verification step, independent of traditional criteria, to identify systematic misalignments in protein alignments. Two non-parametric statistics are shown to be somewhat insensitive to misalignment errors, providing increased confidence in contact prediction when analyzing alignments with erroneous regions because of an emphasis on they emphasize pairwise covariation over group covariation.

## Introduction

Two or more variable positions in a protein may coadapt to conserve interactions needed for proper structure or function [1]–[3]. Strong covariation between pairs of positions is taken to indicate the presence of coadaptation events which are maintained in the alignment as coevolution. It is often assumed that coadapted residues contact each other in the folded protein structure [1], [4]–[6], thus the proportion of putative coadapted positions in contact is often used to benchmark covariation methods. This assumption is not bidirectional, only a small proportion of contacting sites are thought to coevolve strongly [1], [3]. Furthermore, pairs that are very close in sequence are trivially in contact and thus are disregarded when evaluating covariation statistics.

Covariation statistics are often used to aid in residue contact identification, *de novo* protein structure prediction and structure-function analysis [7]–[11]. Indeed, the first step in many structure prediction algorithms is a multiple sequence alignment followed by some sort of covariation measure. Even predictions with modest accuracies are helpful because they restrict the positions to be examined. Standard benchmarks for covariation accuracy measure the fraction of covarying amino acid pairs that are in contact. There are a large number of methods to identify covarying positions [12] in part because some methods work better on certain alignments than on others. Many groups have observed high covariation between residues close in sequence—leading to the belief that two or more positions can only be identified as coevolving if they are some minimum distance apart in sequence. However, little attention is paid to the overall problem that there is no consensus as to the characteristics of truly covarying positions. This results in a counter-intuitive situation where contact is used as a proxy for covariation for benchmarking purposes, but traditional measures like sensitivity and specificity of contact prediction are not very meaningful because only some contacting pairs covary.

Atchley et al. [13], [14] suggested that covariation observed between positions 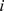 and 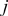 in a protein is composed of a signal from 1) structural or 2) functional constraints, 3) background noise contributed by shared phylogenetic ancestry, and 4) stochastic events. Thus, the structural and functional signal is superimposed on the background noise contributed by phylogeny and by random processes. As implied by this model there are several intrinsic limitations to detecting coevolution between amino acid positions in protein families. First, the sequence alignments must contain sufficient sequences with enough variation for the signal to exceed the noise. Estimates of the required number of sequences needed in the alignments for this to be true vary from 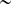
30 [6] to 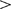
125 [4], [8], [15], [16]. Secondly, all positions in a protein appear to covary because of their shared ancestry, and this signal is the only systematic source of covariation for the vast majority of position pairs [6], [14], [17]. We recently showed that the phylogenetic signal was similar for all positions in a protein family and that it could be estimated as the product of the average covariation of positions 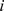 and 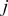 with all other positions [17]. This resulted in ‘product-corrected Mutual Information’ *MIp* and its transform, *Zp*, which was much more sensitive and specific than other previous non-parametric methods.

The goal of multiple sequence alignment is to place residues from each sequence in the protein family at homologous positions in the final sequence alignment. The multiple sequence alignment for a given protein family is usually different in the various standard collections of protein families and the disagreement between protein datasets demonstrates that all multiple sequence alignment methods produce errors. Furthermore, the placement of a gap in the protein family is an explicit acknowledgement that no homologous position exists for one or more members of the protein family. The difficulty in generating a sequence alignment is highlighted by the observation that even structure-based alignment methods disagree [18], [19]. As one example, structure-based alignment algorithms are susceptible to shift error [18], meaning that positions in the structure alignment are not orthologous despite the fact that much of the secondary structural elements seem to overlap between aligned structures.

We observed that the same protein family often gave different numbers of covarying positions when alignments were from different sources even if the alignments contained comparable numbers of sequences. We also found that alignments generated without structural information identified fewer pairs in contact in the folded protein compared to alignments generated with structural information. These observations suggested that the quality of the alignment made a large contribution to the background covariation signal. This observation is supported by Wong et al. [20] who found that alignments of the same sequence dataset by different methods lead to different conclusions in comparative genomics studies.

Here we examine the effect of systematic misalignment on covariation scores. We demonstrate that alignment errors lead to incorrect conclusions about covariation and conservation. We show that *Zp* can be used to identify systematic misalignments in protein families. Furthermore, we show that new statistics 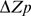 and *Zpx* are relatively insensitive to systematic alignment errors, and are especially effective at identifying pairwise covarying residues. Significantly, these two corrections identify substantially different populations of covarying pairs with similar accuracy.

## Results

### Systematic sources of error

Many commonly-used multiple sequence alignment programs use the progressive sequence alignment strategy in which the alignment and the locations of insertions and deletions are permanently fixed in the growing alignment [21]. An alternative method is structure-based multiple sequence alignment which aligns three-dimensional protein structures and then aligns sequences progressively to the initial structure alignment [22]. Both structure-based and progressive methods systematically propagate early errors through the alignment. Another alignment strategy is iterative alignment, where progressive alignments are built and then iteratively refined to attempt to remove errors that are introduced in the growing progressive alignment [23]. The phylogeny-aware strategy attempts to minimize systematic errors by preventing incorrect gap placement [24]. While it is clear that each alignment strategy is susceptible to varying types and amounts of systematic error, we began by approximating it by using a simple experiment to estimate its effects.

**Figure 1:**
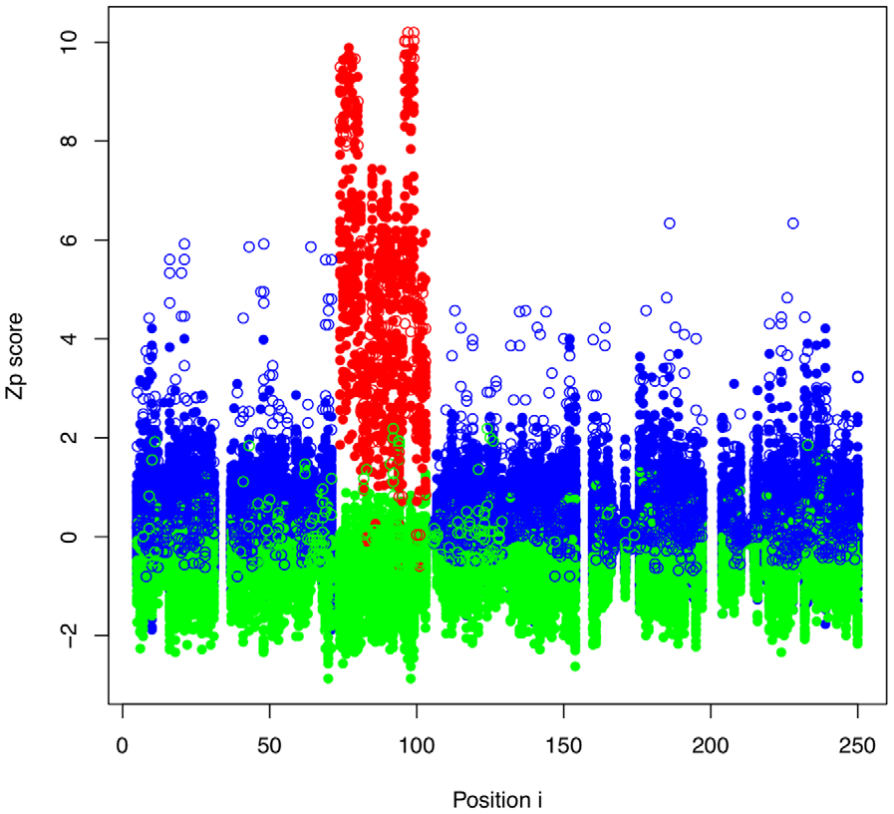
Misalignments cause increased covariation scores. All pairwise *Zp* scores are shown by position in a triosephosphate isomerase alignment which contains a synthetic systematic misalignment in the 4th ungapped segment. Pairs where both positions are from within the misaligned segment are shown in red. Positions where both positions are from outside the misaligned segment are shown in blue. Positions where one position is within and one position is outside the misaligned segment are shown in green. Positions in contact are represented by white-filled circles, positions not in contact are represented by solid colours. The misalignment was made by shifting 20% of sequences in the red-highlighted region by one position to the left or right. Intra-misalignment pairs (red) have higher *Zp* scores and inter-misalignment pairs (green) have lower *Zp* scores when compared to the normal pair distribution (blue).

The impact of systematic errors on the estimation of covariation was tested in an alignment of triosephosphate isomerase by randomly shifting a fraction of the sequences in one aligned segment left or right by 1 residue. We chose to shift between 0 and 30% of the sequences within the selected segment positions—a range chosen because the commonly-used multiple sequence alignment programs have between 70% and 80% accuracy [21]. Figure 1 shows that the *Zp* signal increased if both positions in a pair were in the misaligned segment (red) and decreased if one of the positions in the pair was in the misaligned segment and the other was outside the segment (green) when compared with the pairs unaffected by the misalignment (blue).

**Figure 2:**
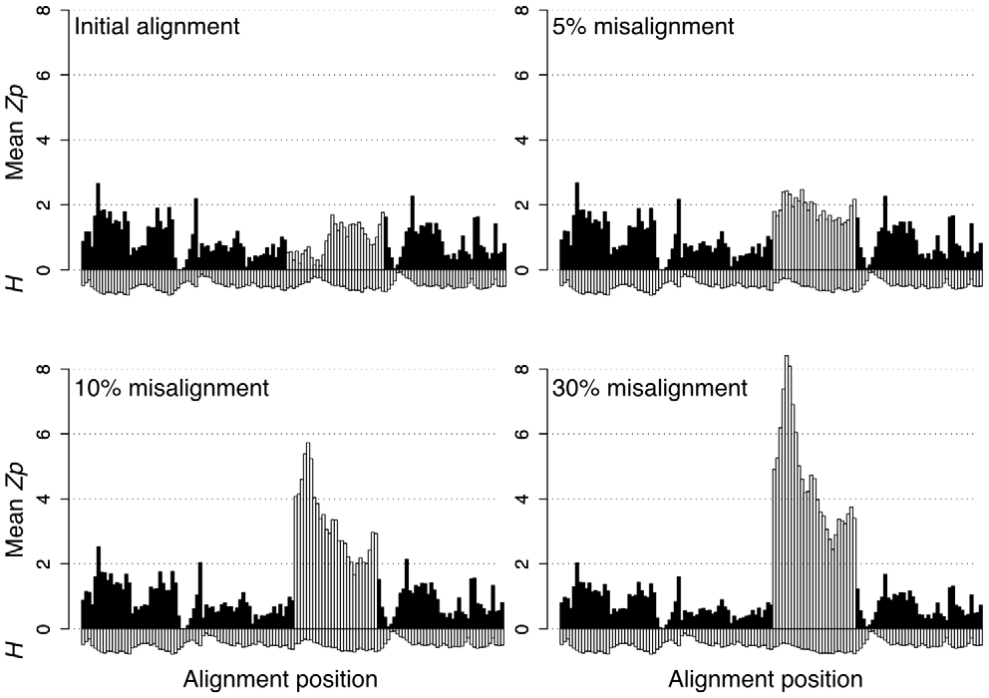
Systematically misaligned regions have high local *Zp* values. The plots show the mean *Zp* score for all pairs of positions in overlapping 6-residue windows versus the window start position. The light bars show the segment of the alignment that was systematically misaligned with the fraction of misaligned sequences indicated. The mean entropy (*H*) of the positions in the same window multiplied by −1 is shown below.

The increased *Zp* values were not distributed evenly among all positions in the alignment (Fig. 1, 2). Figure 1 shows that misaligned pairs have a marked increase in *Zp* score with other residues in the misaligned region. There are several interesting features of such misaligned families: 1) Contacting pairs of positions in the properly aligned regions tend to be assigned *Zp* scores that are much higher than the mean, but often not large enough to stand out against the misaligned region. However, there is often a large difference between the score of a contacting pair and the next-highest score. 2) Noncontacting pairs tend to have small differences between consecutive scores. 3) High-scoring pairs due to misalignment tend to cluster together. Thus we introduce 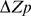, a relative *Zp* measure (Materials and Methods), to compensate for high-scoring noncontacting pairs due to misalignment.

Figure 2 shows a plot of the mean *Zp* score between all pairs of positions in a 6-residue window for a structure-guided alignment of triosephosphate isomerase. The first third of the misaligned segment was highly conserved and had very low *Zp* scores, the remainder was highly entropic and had higher initial *Zp* scores. The systematic misalignment of even 5% of the sequences resulted in a dramatic increase in local *Zp* values for the conserved but not for the non-conserved portion of the segment (Fig. 2). This effect was even more pronounced when larger fractions of the multiple sequence alignment were misaligned. We concluded that systematic sequence misalignment resulted in a characteristic pattern of elevated local *Zp* scores which was most extreme for conserved segments.

**Figure 3:**
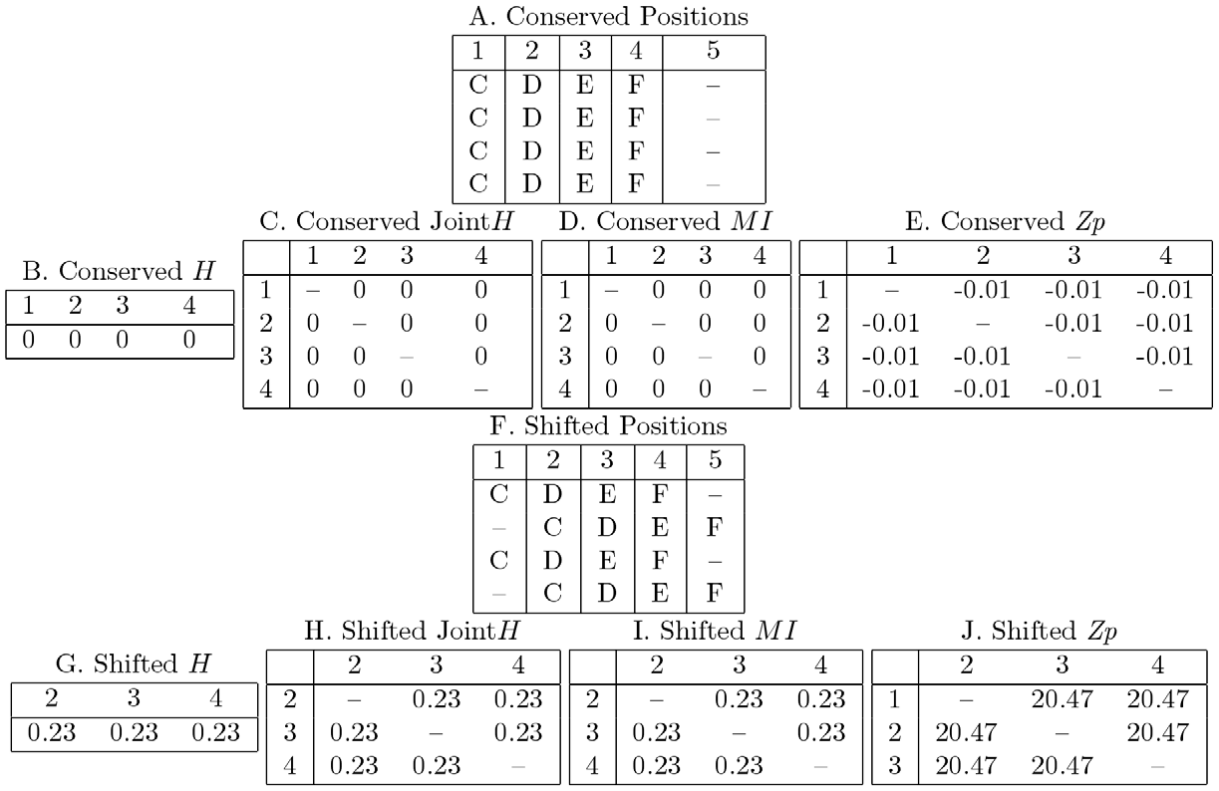
Positions with shift error have high markedly increased covariation scores. When positions 1, 2, 3, and 4 are aligned correctly (A) the positions are conserved and thus there is no entropy (B), joint entropy (C) and therefore no mutual information (D) between the positions. *Zp* was calculated by replicating and attaching the sequences in (A) and (D) to the N-terminus of a triosephosphate isomerase alignment such that every sequence began with the four-residue insertion and gap at the N terminus. The *Zp* scores of the conserved positions fell below the significance threshold of 4.5 (E). The alignment was altered to simulate worst-case shift error; half the sequences were shifted one position to the right (F). These positions have the highest possible entropy (G), joint entropy (H), and mutual information (I) scores for a position with only two residues. As with the conserved alignment, the shifted alignment was inserted at the N-terminus of an alignment of triosephosphate isomerase, such that half the sequences contained a gap after the four residues and the other half contained a gap before the four residues. The resulting *Zp* scores are well above the threshold of 4.5 (J).

The effect of systematic misalignments on the underlying information theoretic values is shown in Figure 3. We consider an arbitrary four residue sequence where each position is conserved (panel A). Each position in this alignment contains no entropy meaning that the residue at that position is certain. Because there is no variation, there cannot be any covariation and thus the values for any covariation statistic is 0. However, when half of the sequences are shifted to the right by one residue, the entropy and joint entropy of the positions increase. The reason that positions 2, 3, and 4 covary is easy to understand intuitively: if you are given the alignment and the identity of a residue at one position, you know the identity of the other two with 100% confidence. It is tempting to think that this effect is simply due to the increase in entropy at each position; this is demonstrably untrue as the effect is still visible when using the covariation statistic *Zp*, which does not correlate with entropy [17].

To demonstrate the increase in *Zp*, the 5-position misaligned block in Figure 3 was attached to the N-terminus of a triosephosephate isomerase alignment so *Zp* could be estimated. The covariation in the misaligned region can not be due to shared ancestry, but rather is an entirely synthetic side-effect of the alignment process and thus the phylogeny correction of *Zp* does not compensate for it. The result is a *Zp* score much higher than the 4.5 cutoff judged to be significant [17]. This effect is analogous to any position which contains information, but with a moderately decreased effect. The increase in covariation is, in fact, due to the proportionally larger increase in positional entropy to joint entropy which is localized only to the misaligned positions.

Little and Chen [25] showed that the covariation statistic, 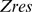, was capable of high accuracy when predicting contacting pairs of positions. 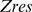 emphasizes pairs of positions which covary strongly relative to the covariation distribution of positions involved, rather than the entire *Zp* distribution. We investigated whether 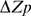 and 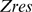 predict contacts with high accuracy because of insensitivity to misalignment. However, we used *Zpx*, a variation on 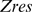 which is calculated more efficiently but is virtually identical (Methods S1).

**Figure 4:**
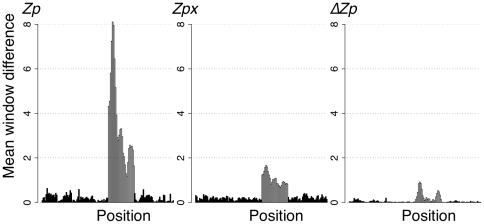
*ΔZp* and *Zpx* are less affected by sequence misalignments than *Zp*. The difference between the mean block scores (*Zp*, *Zpx*, 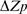 ) for the reference alignment and the alignment containing 30% misalignment is plotted for each measure.

Figure 4 shows the difference across a 6-residue window in mean *Zp*, 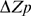, and *Zpx* (calculated as in Materials and Methods) values between the initial alignment and an alignment where an interior segment was systematically misaligned by 30%. Here, positions in the alignment were found to have mean local *Zp* scores that were up to 60-fold greater than the mean local value for the initial alignment (Figure 2 - Initial alignment vs. 30% misalignment). In contrast, the difference scores in 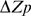 and *Zpx* were much smaller in the misaligned region. Thus, we predicted that high local *Zp* values may be useful to detect misaligned segments in protein families and that 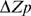 and *Zpx* may be insensitive to misaligned segments.

### Systematic misalignments in CDD

The above analysis on modeled data suggests that if systematically misaligned protein families exist in popular datasets, they might have segments that display high local *Zp* values. The Conserved Domain Database (CDD) [26] was examined for protein families that contained aligned segments displaying elevated mean *Zp* values in 6-residue windows; a number were identified that had 5 or more 6-residue windows with mean local *Zp* scores 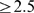 (Table S1). cd00300, the alignment for L-lactate dehydrogenases which is shown in Figure S1A, is one example of an alignment identified to contain systematic misalignments. Examination of the alignment found two sub-populations of sequences that did not fit the overall alignment consensus in these regions (Table S2). One population included sequences that were misaligned in the central portion of the alignment; these were found to be malate dehydrogenase sequences. The second population was composed of partial sequences that were stretched to fit the overall alignment model. Furthermore, the ungapped segments of the structure alignment were placed incorrectly. Removal of both classes of sequence as well as correcting the structure alignment resulted in a more uniform mean local *Zp* as shown in Figure S1B. Interestingly, the residues near the central catalytic core region were much more conserved when the malate dehydrogenase sequences were removed from the alignment. As expected from the modeled data, the contamination of the initial alignment by the paralogous malate dehydrogenase protein family increases *Zp* scores at the conserved active site.

**Figure 5:**
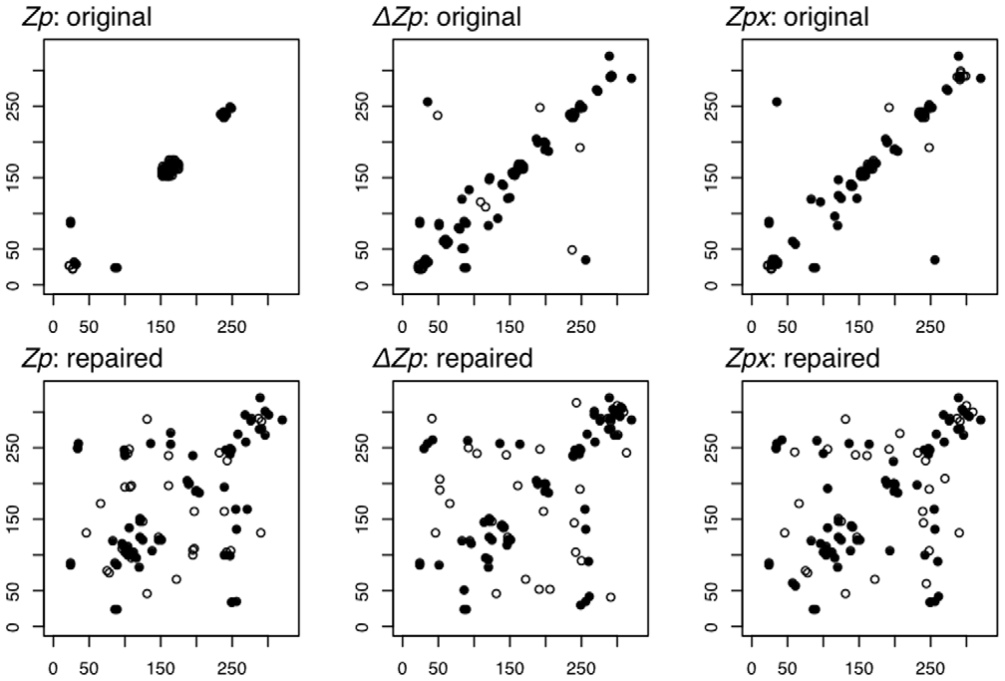
Predicted contact map shows repaired cd00300 alignment is more informative than the original. The top 50 highest *Zp*, 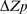, and *Zpx* values were plotted on the 2-D map of the original cd00300 alignment (top) which contains systematic misalignments and the repaired version of cd00300 (bottom) with many misalignments removed. The data is displayed as a predicted contact map where black-filled circles are pairs in contact and white-filled circles are pairs not in contact. The majority of high-scoring *Zp* pairs in the original alignment are uninformative local contacts located in a major region of misalignment (top left). The contact maps of 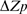 and *Zpx* cover much more of secondary and tertiary structure in both the original and repaired alignments.

Systematic misalignment errors have a dramatic effect on the predictions of a covariance method. The set of predicted covarying pairs are often visualized as a contact map, a two dimensional array where the secondary and tertiary structure of the protein are visible [27]. Because *Zp* is sensitive to systematic misalignment errors, the contact map produced from the original cd00300 alignment contains predictions centered around the sites of misalignment and contains no useful structural information as shown in Figure 5-*Zp*:original. In contrast, 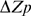 and *Zpx* produce more informative contact maps than *Zp* even with systematically misaligned data; however, the contact maps are largely composed of local contacts covering the secondary structure of the protein (Fig. 5-original). When the repaired alignment was used, the contact maps of all three measures show predictions across the length of the protein encompassing both secondary and tertiary structure (Fig. 5-repaired). We conclude that the corrected alignment was more informative than the initial alignment for contact prediction.

We next measured the mean number of pairs in contact between the pair of positions with the 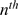 highest or better *MI*, *Zp*, 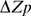 or *Zpx* values using the set of alignments in the CDD that met the minimum criteria, outlined in the Materials and Methods; these criteria are established as requirements to accurately identify contacting pairs in protein families. From these families, those that possessed 5 or more 6-residue segments with mean local *Zp* values 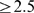 were selected. There were 16 such families that met the criteria for making contact predictions. These alignments are referred to as the ‘worst CDD’ because they are likely to contain systematic misalignments. The 84 protein families which were not included in the worst alignments set, which also met criteria for presence of covariation (Materials and Methods) are referred to as the ‘best CDD’ dataset. We also examined 5 highly curated protein families that were aligned using the Cn3D program [22] with multiple structural lines of evidence to support the inclusion of each sequence in the alignment (Materials and Methods).

**Figure 6:**
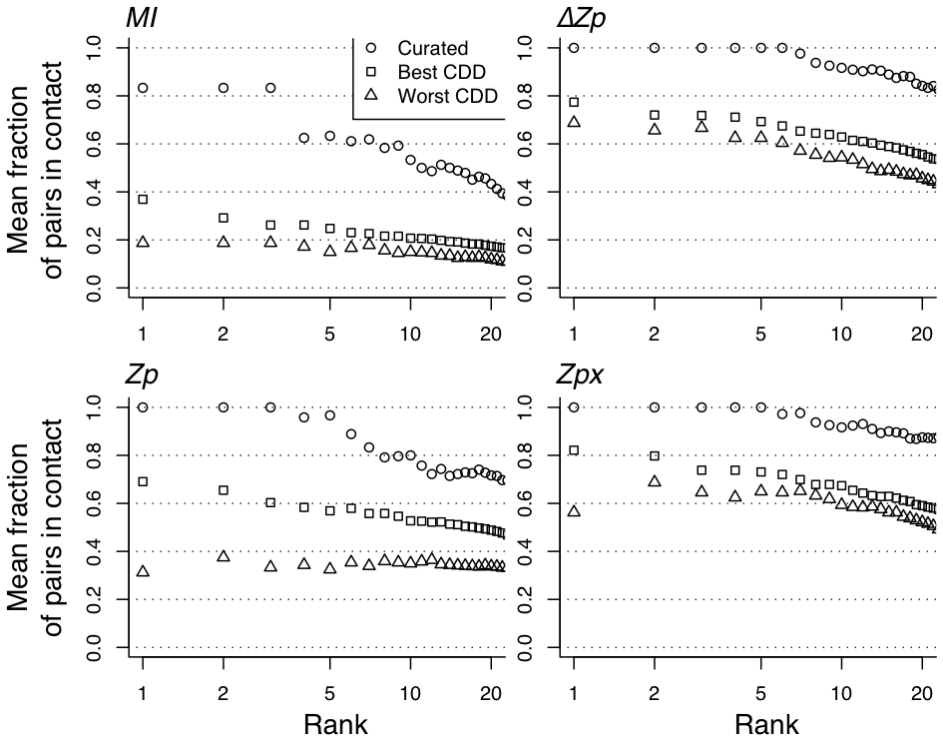
The effect of alignment quality on contact identification. All methods identify many contacts in the curated dataset. Plotted here is the mean fraction of pairs in contact of the top 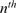 ranked pairs (up to 20) for the Curated, Best CDD, and Worst CDD datasets using *MI*, *Zp*, 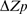, and *Zpx*. The Curated dataset contains ideal alignments that are hand-curated for accuracy. Best CDD contains alignments which are unlikely to contain systematic misalignments. Worst CDD contains alignments which are likely to contain systematic misalignments. Only pairs 10 or more positions apart in sequence are included to prevent proximity in sequence from biasing results.

The circles in Figure 6 show that all four covariation measures were able to identify many contacting pairs of positions in the curated alignments. *MI* was the worst performing measure, but the top 3 pairs were in contact in 4 of the 5 curated alignments. *Zp* was much better than *MI*, and 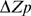 and *Zpx* outperformed *Zp*. The 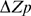 and *Zpx* measures had similar accuracies; the top 7 highest scoring pairs of both 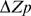 and *Zpx* were in contact and the top 20 pairs of each had a 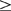
80% likelihood of contact.

All four methods performed much worse in the CDD-based datasets. Again 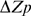 and *Zpx* performed similarly and both were superior to *Zp*. However, *Zp* and *MI* identified very few positions in contact in the ‘worst’ alignments, and the highest scoring pair was no more likely to be in contact than the n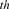-best scoring pair. We suggest that the large disparity between contact prediction accuracy of *Zp* on the best and worst datasets is because *Zp* is sensitivite to systematic misalignments present in the ‘worst’ dataset while 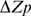 and *Zpx* are not.

### Comparison of sensitivity

We were interested in the sensitivites of *Zp*, 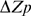, and *Zpx*. The sensitivity of covariation methods is affected by the number of sequences [15], the number of positions in the alignment [17] and the structure used as the reference. The sensitivity of each method was determined by assessing the number of contacting pairs found at given likelihoods of contact, and is shown in Table S3 for contact likelihoods between 50% and 90%. All three methods identified over 17 pairs of positions per protein family with a contact likelihood of 50%. The number of pairs identified dropped off dramatically as the contact likelihood increased; *Zp* found an average of 4.4 pairs, 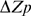 found an average of 5.6 pairs and *Zpx* identified 6.5 pairs at 90% contact likelihood. We conclude that *Zpx* is more sensitive than the other measures, but that no measure vastly outperforms any other.

**Figure 7:**
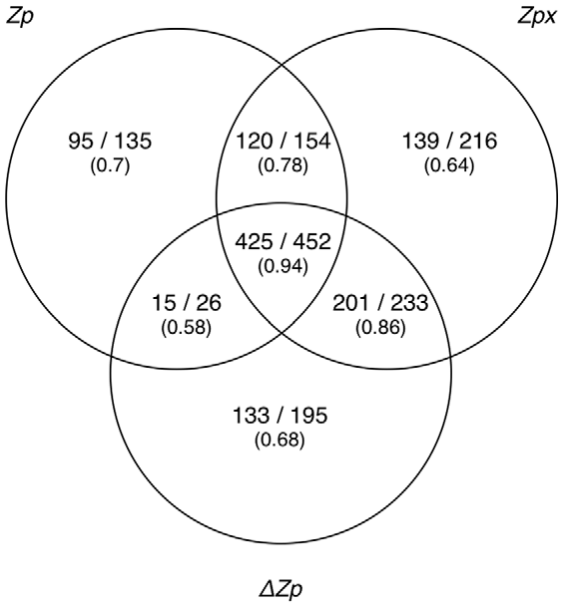
*Zp*, *ΔZp*, and *Zpx* find different subsets of contacting positions. Venn diagram of pairs identified at 80% contact likelihood cutoff shown as number in contact vs. total predictions. Only pairs 10 residues or more apart in sequence were considered to prevent proximity in sequence from biasing results.

We wanted to know if the three methods were identifying the same set or a different set of contacting pairs. We identified alignments in the CDD where 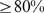 of the pairs found were in contact in each alignment (Materials and Methods, Table S4). *Zp* identified 767 pairs of which 85% were in contact, 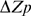 identified 906 (85% contacting) and *Zpx* identified 1055 pairs (84% contacting). The overlap in the 1411 total pairs is shown in Figure 7, with the proportion of pairs in contact given below each measure. Several observations can be made. First, no method identified all pairs found by any other method; *Zpx*, 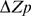 and *Zp* identified 75%, 64% and 54% of the total pairs with 15%, 14% and 10% of the pairs being unique to each method. The pairs most likely to be in contact were pairs identified by both 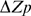 and *Zpx*. These 685 pairs composed 49% of the total pairs identified with 626 in contact (91% contacting). We conclude that pairs identified by both 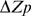 and *Zpx* were more likely to be in contact than if either was used by itself.

### *ΔZp* and *Zpx* emphasize pairwise covariation

We were interested why 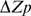 and *Zpx* were so effective at identifying contacting pairs. We examined this by modeling group coevolution with the simple model of *in silico* evolution described in the Materials and Methods and generated 10 independent alignments where the size of the coevolving group varied between 0 (no covariation) and 10, and the probability of coevolution was varied in increments between 0 and 0.95. These model alignments were used to examine the relationship between group size and the covariation score for each measure.

**Figure 8:**
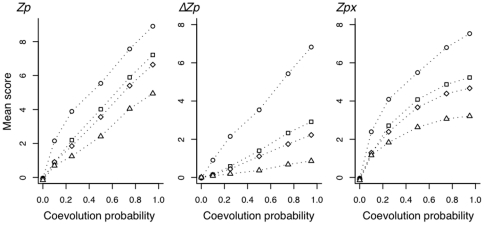
The effect of covariation probability and covarying group size on covariation measures. Sequences were evolved with each residue having a fixed probability of changing to another residue per time step as described in the Materials and Methods. Positions were placed in groups of 2 (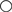), 4 (□), 5 (◊), or 10 (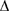) and constrained to coevolve with likelihoods of 0.1, 0.25, 0.5, 0.75 and 0.95. For example, if the group size was 4 and the likelihood of coevolution was 0.25, then if the residue of one member of the group was changed during the time step, then each of the other three members of the group was allowed to change to another residue with the fixed probability given on the X axis. The Y axis shows the mean score for each group size and each probability for 10 replicate *in silico* evolution runs.

Figure 8 shows the effect of group size and coevolution probability on the mean values for the three statistics. If we examine the mean values for the extreme case where coevolution occurs at the maximum probability, we see that in all 3 methods the pairwise coevolving positions (ie. group size = 2) have much higher mean values than do instances where coevolution occurs in groups of 10. This effect is less pronounced for the intermediate group sizes of 4 or 5. In the case of *Zp*, residues that coevolve with an intermediate group size attain mean scores greater than 6, which is close to the mean 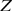 score for coevolving pairs of positions. However, both 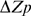 and *Zpx* show markedly lower mean scores for the intermediate sized groups of coevolving positions than for pairwise coevolving positions. The effect is similar at lower coevolution probabilities for all three methods, although it is non-linear for *Zpx*. We conclude that the ability of 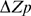 and *Zpx* to emphasize the effect of pairwise covariation at the expense of group covariation explains in part why these two methods identify contacting pairs with greater sensitivity and specificity than other non-parametric methods.

## Discussion

It is assumed that the covariation signal derived from structural and functional constraints is superimposed on the phylogenetic and stochastic signal [14]. This idea has led several groups to make the assumption that if a method identifies some structural covariation as indicated by contacting pairs, then other pairs with equivalent or higher scores *not in contact* must be caused by functional constraints [5], [8], [11]. However, since the true coevolving pairs in an alignment are unknown [14], [28], the alternative explanation is that some of these pairs may be false positive identifications. Here we show that a strong covariation signal can be caused by alignment error, potentially leading to false positive predictions. Specifically, covariation analysis of cd00300 would result in incorrect assignment of both conserved and covarying residues and thus an incorrect understanding of the protein.

Local covariation increases with misalignment proportionally to the amount of conservation at a position and inversely proportional to the amount of entropy. Covariation can be understood as proportionally high positional entropy relative to low joint entropy. This is related to the underlying information-theoretic values outlined in figure 3. Positions with low conservation are less susceptible to the local covariation effect because they already contain many of the possible residues making the proportional increase of entropy to joint-entropy less significant. Similarly, misaligning random sequences has a smaller effect than sequences that are misaligned together as a clade. If sequences are misaligned as a clade, the increase in joint-entropy is proportionally smaller because the sequences are related and aligned together which causes a larger increase in local covariation. The test to generate figures 1 and 2 is very conservative since the selection and shift are both done randomly. Conversely any algorithm which uses a phylogenetic tree to build the alignment will be susceptible to hierarchical clustering of misaligned sequences, which are easier to detect.

It is worth noting that the ability to detect misalignments is unique to *Zp* when compared to other statistics outlined in this manuscript. 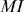 does not identify misalignments because background covariation signal is too high. The 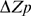 and *Zpx* statistics do not identify mistalignments because they filter out the misalignment covariation signal. *Zp* works because it transforms the covariation values based on the assumption that the background covariation is due to shared phylogeny and relative entropy — an assumption that is explicitly violated when misalignments are introduced.

The increased local covariation methods outlined in this paper have already been critical to the completion of two publications. In one [29], Gloor et al. used local covariation (as in Figure 2) to improve a structure-based sequence alignment of phosphoglycerate kinase. As outlined below, increased local covariation was the crucial tool that identified regions in the alignment which were likely to produce false positive results. In the second [30], Kleinstiver et al. used increased local covariation to validate an alignment of the GIY-YIG homing endonuclease I-Bmol, and prevented contamination by paralogous sequences. Furthermore, covariation statistics *Zp*, 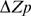, and *Zpx* were used to identify new structurally and functionally important pairs of residues. These successes demonstrate the effectiveness of increased local covariation and the new covariation statistics.

We found that flaws in the alignments themselves often result in positions having high covariation scores because of systematic misalignments. Since systematic misalignments involve several positions that are close in sequence (eg. cd00300), this could explain some of the group covarying positions that have been seen by many investigators, eg. [5], [6]. We suggest that evidence of group covariation between residues close in sequence be investigated carefully. For example, Gloor et al. found that increased local *Zp* identified two regions of phosphoglycerate kinase which contained subclusters of residues found in completely different environments [29]; structurally-conserved segments were either exposed to solvent or buried because of the replacement of a nearby alpha helix in some structures with a beta strand in others.

The logic of building an alignment is partially circular: alignments are built in part by maximizing sequence conservation, but then are used to find conserved positions which are, in turn, identified as important. While structure-based methods are often used as the benchmark and standard for protein alignments [31], Löytynoja and Goldman [24] showed that structure-based alignments are not as reliable as expected for genome annotation. Similarly, we found that some structure-based protein alignments are inappropriate for covariation analysis and, as noted above, that *Zp* can identify misaligned regions. We found that markedly elevated local *Zp* values were the hallmark of misaligned regions and suggest that the investigator proceeds with caution during the analysis of positions showing this pattern of covariation. If an investigator draws conclusions from an alignment which has not been examined with increased local covariation, there is an increased risk of drawing erroneous conclusions from the alignment.

Finally, we demonstrated that *Zpx* and 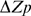 are relatively insensitive to sequence misalignments explaining the increased sensitivity and selectivity of these methods to identify contacting pairs. The ability to identify misaligned segments coupled with the use of measures insensitive to misalignment reduces the risk of systematic misalignment and provides opportunities to correct and re-analyze the alignment. *Zpx* and 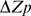 identified different subpopulations of pairs, which implies that neither method is, as yet, optimal. We find it interesting that both modifications use the independent covariation signal from positions 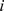 and 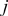 to derive the final statistic, suggesting that the relative covariation signal of each position is informative.

The sensitivity of *Zp* to systematic alignment errors can be exploited to identify regions of potential misalignment. Covariation provides an independent method for verifying the quality of an alignment and should therefore be especially useful for verifying alignments built on sequence conservation alone. The cd00300 alignment showed that misalignments occur in structure-based alignment datasets; importantly, cd00300 showed that misalignments occur in functionally-important conserved regions. We recommend investigating any incidents of increased local *Zp* (as in Figure 1, 2 or S1) as they may indicate systematic misalignment or an interesting phenomenon causing increased local covariation. We conclude that increased local covariation is an effective guide for improving or validating a multiple sequence alignment and initial observations suggest that mean pairwise *Zp* scores above 2.5 over a window of 6 should be investigated.

Our work shows that the quality of the alignment is critical for correct assignment of pairs of residues in covariation analyses; unfortunately, all alignment methods produce lower-quality covariation predictions when erroneous sequences are included. However, it is impossible to know for certain if all positions in an alignment are assigned correctly even when using state-of-the-art methods. Therefore, we recommend 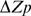 and *Zpx* over other statistics as they provide better contact prediction because they are demonstrably less susceptible to systematic misalignment errors than other covariation measures like *Zp*.

## Materials and Methods

### Modeling Systematic Misalignment

A hand-curated alignment for triosephosphate isomerase was created using Cn3D [22]. An ungapped segment of the alignment was selected to be the artificially misaligned segment. The misaligned segment is highlighted in each figure. Within this segment, each sequence has a 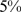, 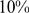, or 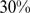 chance of being shifted. Each sequence selected to be misaligned has an equal chance of being shifted one position left or right.

### Alignment curation and criteria for contact prediction

Multiple sequence alignments were extracted from the CDD database downloaded from NCBI on April 17, 2008. They were curated to include only those alignments with at least one structure, more than 125 sequences and 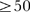
50 ungapped positions in the alignment. Three datasets were benchmarked in Figure 6. ‘Worst CDD’ are a subset of the outlined CDD sequences which are likely to contain misalignments as they contain 5 or more 6-residue segments with mean *Zp* 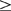
2.5. ‘Best CDD’ contains sequences which are not in the ‘Worst CDD’ dataset, but which also have at least (L/10) values of *Zp* 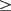
4.5 (where L is the length of the protein). This ensures that the alignment has some covariance information, but makes no judgements about the location of these pairs in sequence or in structure. The ‘Curated’ dataset contains 5 structure-based hand-curated alignments curated according to Dunn et al. [17]. For Figure 7, we identified alignments in the CDD that had 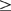
150 sequences, 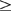
50 non-gapped positions. We curated this set so that each covariance method was able to predict contacting pairs at an accuracy of at least 80%. To ensure the structure used to assign contacting pairs did not bias the results, we used alignments where the covariance methods agreed on which structure was of highest quality. The 100 alignments meeting these criteria are listed in Table S4. When covariation statistics predict contacting pairs, we define contact as any non-hydrogen atom from one residue being within 6 Å of any non-hydrogen atom from the other residue. To prevent proximity in sequence from biasing results, only positions 10 or more apart in sequence are considered when using contact as a benchmark.

### cd00300-based alignments

cd00300 is a structure-based alignment of lactate dehydrogenase from CDD. The original cd00300 dataset is composed of the sequences in this alignment. The repaired cd00300 dataset has 13 sequences removed because of alignment issues (annotated in Table S2). The original cd00300 alignment is used as it existed in CDD. The repaired version of cd00300 was realigned using Cn3D [22] based on a refined structure alignment based on errors of probable misalignment according to increased local covariance.

### Covariance statistic calculations

Covariance statistics were calculated according to Martin et al. [15], and Dunn et al. [17]. *Zpx* is similar to the 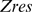 statistic of Little and Chen [25], but simpler to calculate (Methods S1). Positions that contain gaps are not analyzed because gaps violate the assumption of orthology connecting covariation with coadaptation and coevolution (Figure S2).

Mutual Information (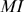) measures the reduction in uncertainty of one variable given information about another variable and was calculated as previously [15]. As shown in equation 1, in the context of protein sequence families it measures the difference between the expected entropy (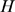) of residues in two columns 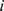 and 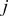 if they were independent against the observed joint entropy, 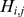.
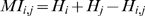
(1)The underlying assumption when calculating *MI* is that all events are independent; this is not true in the context of protein families since, to a first approximation, every position in a protein family shares common ancestry with every other position, and the positions in a gene for a given protein are rarely split by recombination. *MIp*, the product corrected *MI*, estimates the background *MI* signal caused by sequence non-independence [17] as shown in equation 2
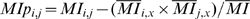
(2)where 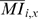 is the mean *MI* of position 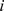 with all other positions and 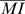 is the overall mean *MI*.

The *MIp* values were converted to *Z* scores since absolute *MIp* values vary somewhat between alignments and because the underlying distribution of *MIp* values approximates a Gaussian distribution in the absence of structural and functional covariation [17]:
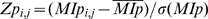
(3)where again 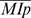 is the mean *MIp* and 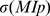 is its standard deviation.

*Zpx* is a modification of Little and Chen's 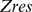 statistic [25] that is based on the residual 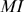 of a linear regression between 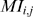 and the mean 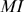 of positions 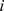 and 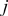, 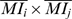. The plot shown in Figure 1A shows that the residual is nearly identical to *MIp* with a slope of 1, an intercept of 0 and an 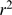 value of 0.9995. 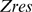 is the product of the *Z* scores derived from the residuals for each individual position [25]. Since the residual and *MIp* are virtually indistinguishable (Methods S1), we substituted the more efficiently calculated *MIp* for the residual in the formula for 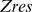 as shown in equation 5 to calculate 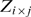.
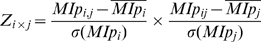
(4)


As expected from the similarity of the underlying statistics, 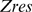 and 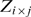 are extremely similar with a slope of 0.9998, an intercept of 0.0005 and an 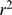 value of 0.9998. Note that the 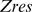 and 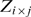 values are the product of the two 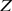 scores, and so these values scale geometrically when compared to 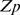. In this study we use *Zpx*, which is the square root of 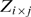 to allow comparison of the 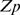 and 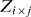 values on similar scales.


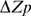
 is a measure of the difference score between successive *Zp* scores at position 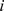. 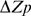 was calculated by placing the *Zp* values of position 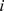 with all other positions, 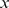, in an ordered list, with the largest *Zp* value first, i.e. 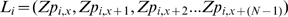, where 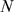 is the number of *Zp* values for position 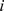. 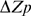 is the sequential difference between list elements scaled by interquartile units as follows:
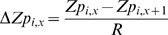
(5)where 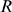 is the interquartile range calculated as 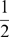 the difference between the 75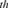 and the 25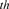 percentile for the data in 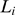. The interquartile range scaling factor and the median provide robust estimates of dispersion and central tendency that compensate for variation in the distribution of *Zp* at each position while making the minimum number of assumptions about the underlying distribution. Thus 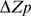 measures how extreme the difference in *Zp* scores is for all pairs of positions, 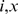.

Since each *Zp* score is a measure of the covariation between two positions, 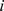 and 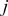, there are two 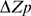 scores for each pair of positions: one is the difference between *Zp* 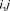 and the next highest score for position 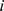, and the other is the difference between *Zp* 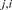 and the next highest scoring position for position 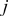. We used the greater of the 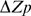 scores.

*MI*, and the derived statistics, *MIp*, 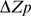 and *Zpx* were calculated only for *ungapped* positions in the multiple sequence alignments. Covariation analysis attempts identify those positions that are coevolving for structural or functional reasons, and as shown in this report, depends upon the precise placement of homologous positions in the alignment.

### Screening for misalignments using increased local MIp

The average 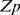 score and average entropy were calculated for all pairs in a ungapped window of width 6. When graphed, high peaks represent increased local 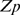. We consider peaks of height 2.5 or higher to be worth investigating, but these could also represent strong covariation due to secondary structure.

### Synthetic coevolution dataset

The data in Figure 8 were generated using the simple model of coevolution described previously [15]. In brief, the initial sequence had substitutions introduced at each position with a probability derived from a uniform probability distribution. Sequences were split or ‘speciated’ with a constant probability. Groups of positions were constrained to coevolve such that if a substitution occurred in one member of the group the remaining members of the group had a substitution introduced with a given probability which ranged between 0.1 to 0.95. Group sizes were varied between 2 and 10. Alignments derived from this method have been shown to recapitulate many properties relevant to coevolution [15].

## Supporting Information

Methods S1 Supplementary figure and text for Material and Methods section.
(0.75 MB PDF)

Figure S1 A plot of local *Zp* values in cd00300, the lactate dehydrogenase superfamily. Panel A shows a histogram of the mean *Zp* value between all pairs of ungapped positions in a 6 residue window. Red and blue bars are positions in the alignment that are adjacent to indels. The mean entropy of the residues multiplied by −1 in the window is plotted in green below. Panel B shows the same plot from an alignment with the malate dehydrogenase sequences and partial sequences (identified in Table S2) removed with a subsequent adjustment of the structure alignment.
(0.03 MB PDF)

Figure S2 Positions containing gaps violate the assumption of positional homology. Strong structural conservation flanks an insertion of two residues in a surface loop. The gap region is highlighted in grey and is two residues long for the shorter sequences and four residues long for the longer sequences. Highlighted in yellow are two alternate hypotheses of the residues which are homologous to the two residues in the shorter sequences. Structurally, it is impossible to determine which two residues are homologous to the shorter gap sequences. Choosing two of the four residues in the insertion region as homologous to the shorter sequence residues will likely introduce error into the alignment.
(0.13 MB PDF)

Table S1 Alignments containing probable systematic misalignments. Table of alignments in CDD which have 5 or more peaks of mean *Zp* score greater than or equal to 2.5 for all pairs of positions over a residue window of width 6.
(0.04 MB PDF)

Table S2 Sequences removed from cd00300 because of poor alignment to structure alignment. Not all possibly erroneous sequences were removed in order to meet the cutoff for minimum number of sequences for covariance methods.
(0.08 MB PDF)

Table S3 Scoring cutoffs for arbitrary accuracy in CDD alignments. CDD alignments were examined and those that contained ≥150 sequences, ≥50 nongapped positions and where each method made at least one true prediction were identified at an accuracy of 80% or higher. There were 100 such protein families and they are identified in Table S4. The cutoff values for each measure at which at least the fraction of pairs identified were in contact is given along with the mean number of pairs identified in each protein family. Previous work had identified a cutoff of 4.5 for *MIp*, and the analysis here suggests that cutoff is appropriate for ∼80% accuracy. Only pairs 10 or more positions apart in sequence are included to prevent proximity in sequence from biasing results.
(0.02 MB PDF)

Table S4 Venn Diagram Alignments. Alignments curated according to Materials and Methods, used to generate Figure 7 and Table S3.
(0.09 MB PDF)
