# Supplementary material for: Identifying and Seeing beyond Multiple Sequence Alignment Errors Using Intra-Molecular Protein Covariation
Source: PLoS One. 2010 Jun 28;5(6):e11082. doi: 10.1371/journal.pone.0011082 (PMC2893159; doi:10.1371/journal.pone.0011082)
Supplement: Methods S1 — Supplementary figure and text for Material and Methods section. (0.75 MB PDF) [file pone.0011082.s001.pdf]

Supplementary Methods

The $Zres$ [Little and Chen(2009)Little and Chen] and $Z_{i \times j}$ statistics were very similar; the plot in Figure 1B shows a slope of 0.9998, an intercept of 0.0005 and an r^2 value of 0.9998. However, since the $Z_{i \times j}$ statistic is a multiplicative Z score, it greatly expands the extreme tail of the distribution and is not easily interpretable. Thus, we chose to use $\sqrt{Z_{i \times j}}$ since this value maintains the same order of scores without unduly over-representing the difference between covarying and non-covarying pairs. We refer to the $\sqrt{Z_{i \times j}}$ as the Zpx score.

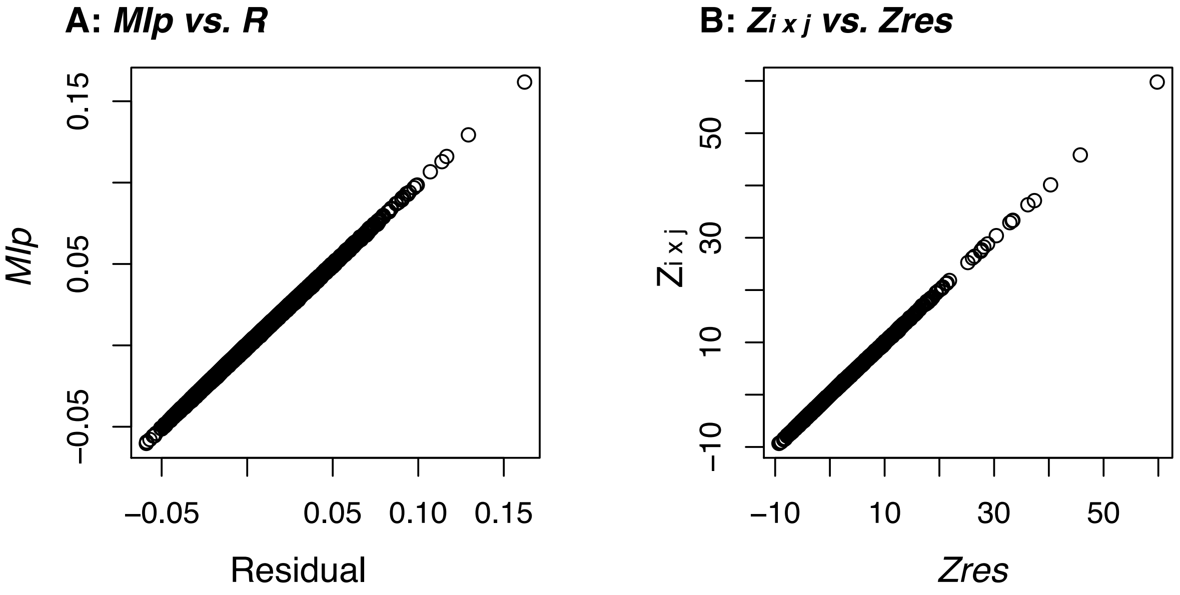

Figure 1: MIp and $ZRes$ are essentially the same statistic. Panel A shows a plot of MIp *vs.* the residual of a linear regression between MI_{ij} and $\overline{MI_i} \times \overline{MI_j}$ [Little and Chen(2009)Little and Chen]. Panel B shows a plot of $Z_{i \times j}$ *vs.* $Zres$. The data were calculated using the repaired lactate dehydrogenase alignment. These statistics were very similar. In A, $r^2 = 0.9994975$ with a slope and intercept of: 1.000 and 6.185e-05. In B, $r^2 = 0.9998498$ with a slope and intercept of 0.9998193, 0.0005206.

References

[Little and Chen(2009)Little and Chen] Little, D. Y. and Chen, L. (2009). Identification of coevolving residues and coevolution potentials emphasizing structure, bond formation and catalytic coordination in protein evolution. *PLoS ONE*, **4**(3), e4762.
