# Supplementary figures and images for: Identifying and Seeing beyond Multiple Sequence Alignment Errors Using Intra-Molecular Protein Covariation
Source: PLoS One. 2010 Jun 28;5(6):e11082. doi: 10.1371/journal.pone.0011082 (PMC2893159; doi:10.1371/journal.pone.0011082)

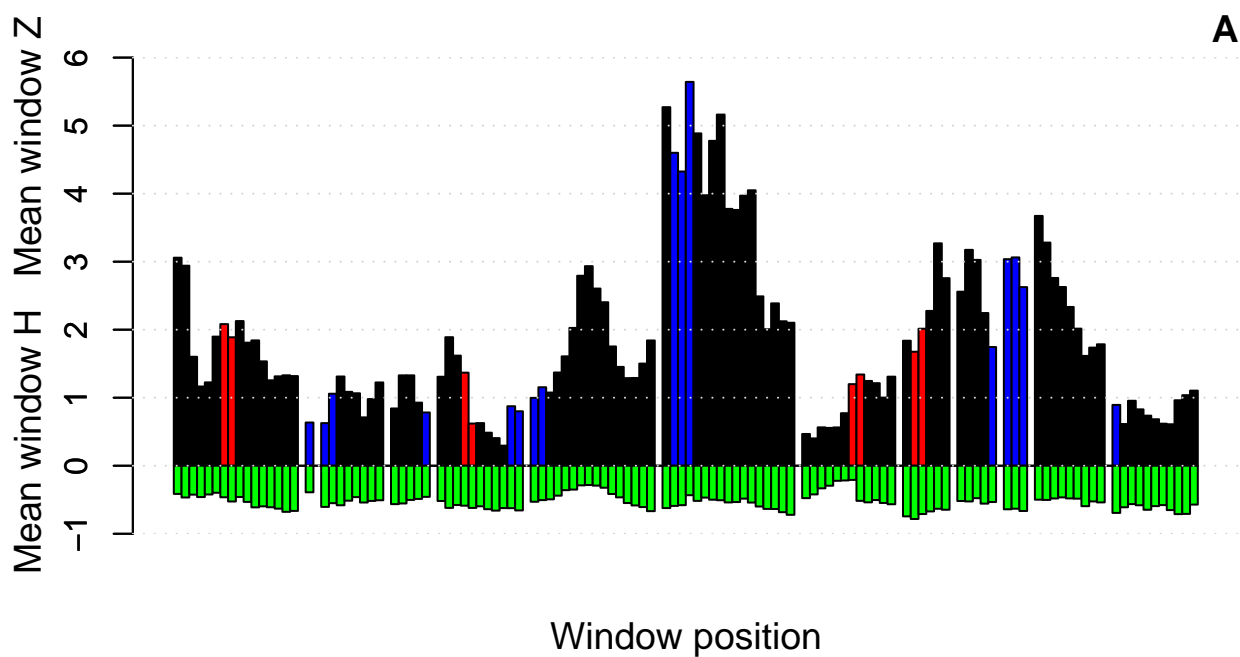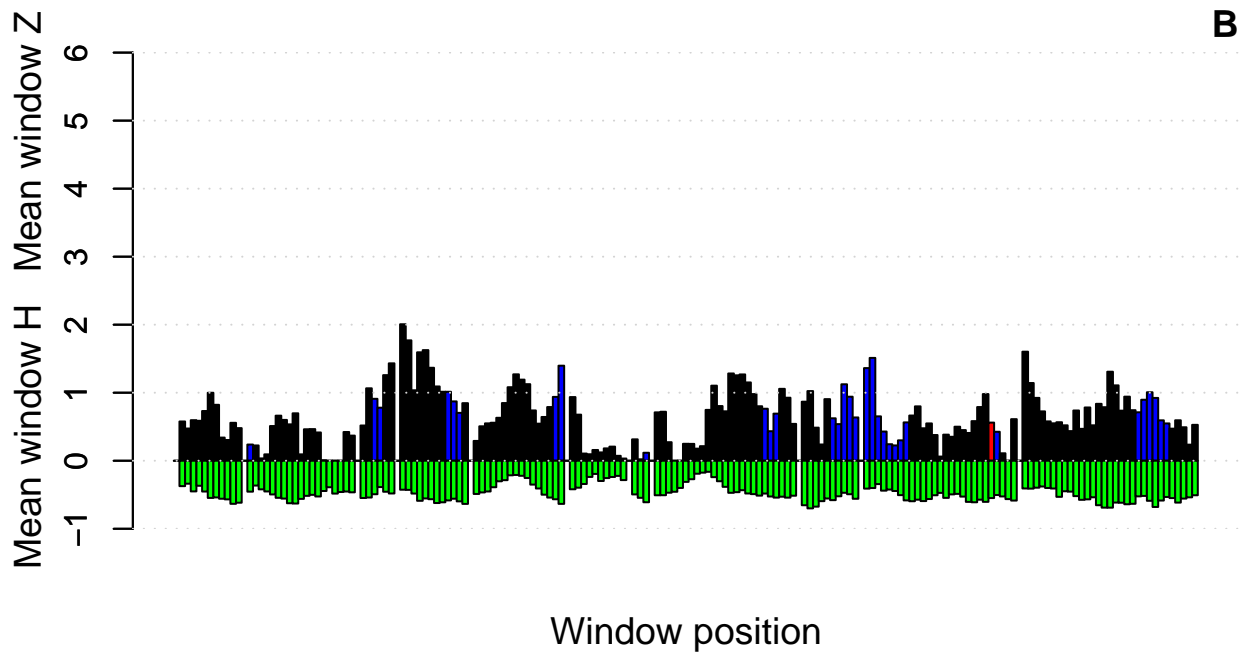

Supplement: Figure S1 — A plot of local Zp values in cd00300, the lactate dehydrogenase superfamily. Panel A shows a histogram of the mean Zp value between all pairs of ungapped positions in a 6 residue window. Red and blue bars are positions in the alignment that are adjacent to indels. The mean entropy of the residues multiplied by −1 in the window is plotted in green below. Panel B shows the same plot from an alignment with the malate dehydrogenase sequences and partial sequences (identified in Table S2) removed with a subsequent adjustment of the structure alignment. (0.03 MB PDF) [file pone.0011082.s002.pdf]

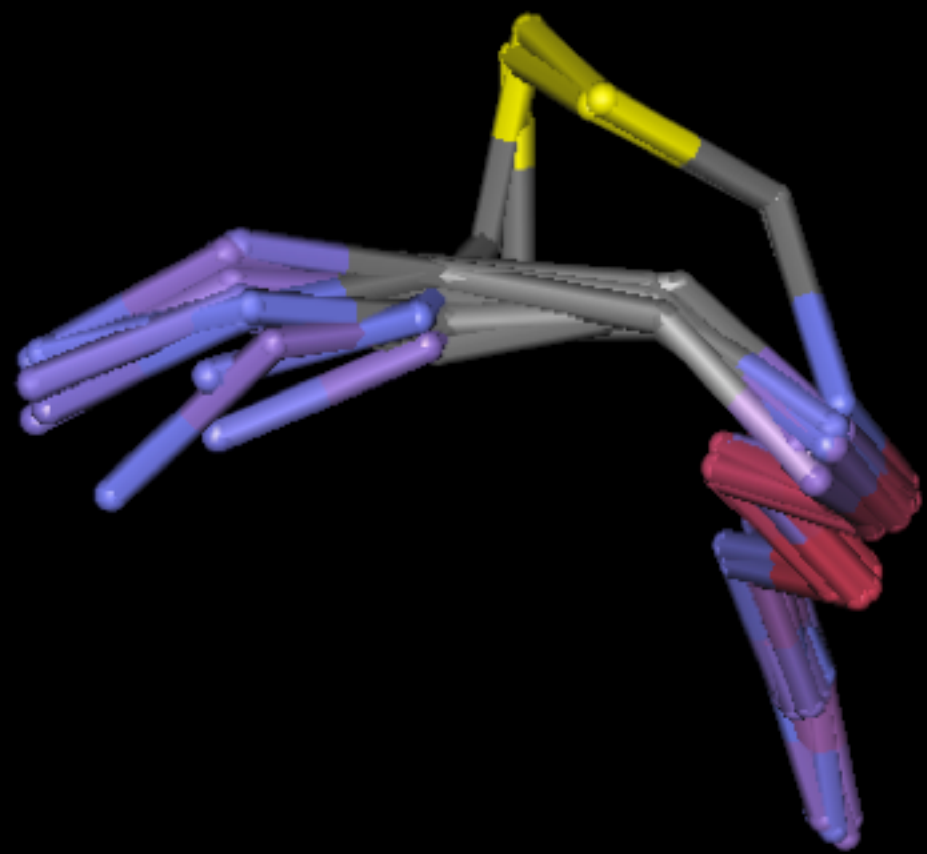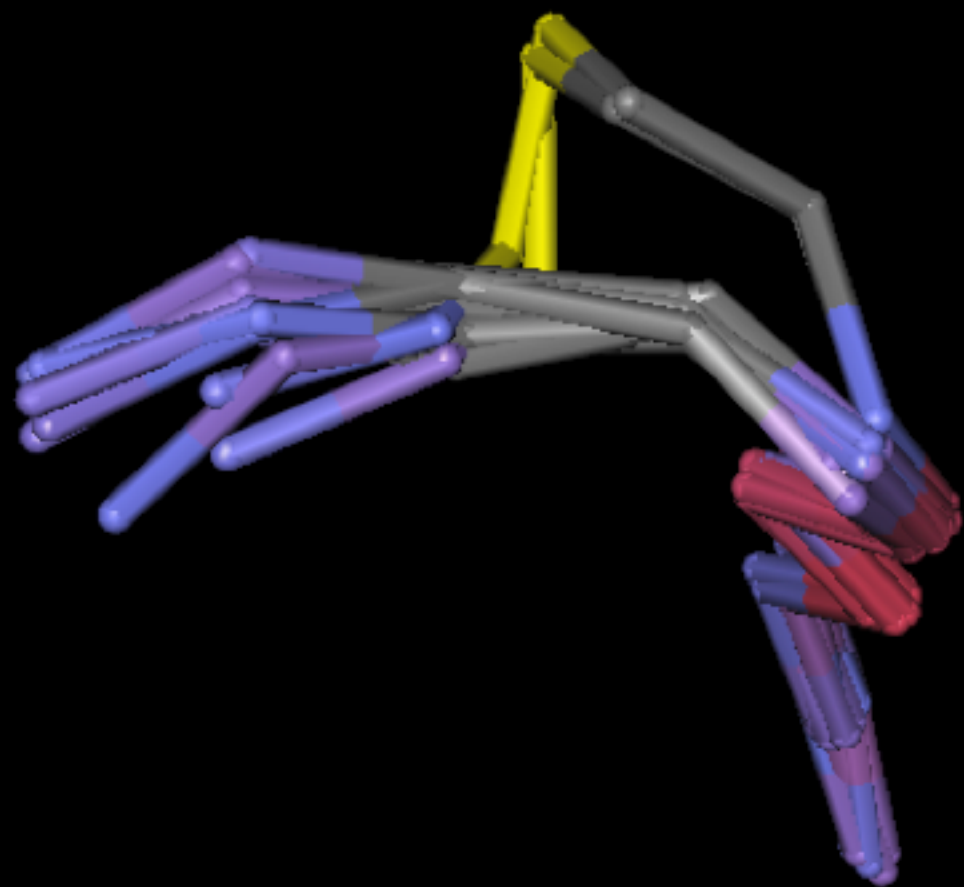

Supplement: Figure S2 — Positions containing gaps violate the assumption of positional homology. Strong structural conservation flanks an insertion of two residues in a surface loop. The gap region is highlighted in grey and is two residues long for the shorter sequences and four residues long for the longer sequences. Highlighted in yellow are two alternate hypotheses of the residues which are homologous to the two residues in the shorter sequences. Structurally, it is impossible to determine which two residues are homologous to the shorter gap sequences. Choosing two of the four residues in the insertion region as homologous to the shorter sequence residues will likely introduce error into the alignment. (0.13 MB PDF) [file pone.0011082.s003.pdf]
