# Supplementary material for: Identifying and Seeing beyond Multiple Sequence Alignment Errors Using Intra-Molecular Protein Covariation
Source: PLoS One. 2010 Jun 28;5(6):e11082. doi: 10.1371/journal.pone.0011082 (PMC2893159; doi:10.1371/journal.pone.0011082)
Supplement: Table S1 — Alignments containing probable systematic misalignments. Table of alignments in CDD which have 5 or more peaks of mean Zp score greater than or equal to 2.5 for all pairs of positions over a residue window of width 6. (0.04 MB PDF) [file pone.0011082.s004.pdf]

alignment	number of windows ≥ 2.5
cd00688	22
pfam07992	9
cd01288	8
cd02889	23
cd00418	13
cd00267	10
cd04322	6
cd00812	5
pfam00501	5
cd02750	6
cd01991	5
cd00751	16
cd00807	7
cd01337	36
cd00817	32
COG0124	5
cd03586	6
cd00046	5
cd04317	10
cd00347	8
pfam03063	5
pfam00310	8
cd01596	20
cd03747	8
pfam00152	7
cd01339	30
cd01967	5
smart00642	7
cd00300	33
pfam00753	15
cd00672	21
pfam00702	10
cd02023	7
cd00818	29
cd00802	19
cd01594	7
cd00771	5
cd00808	5
cd01359	5
cd01914	5
cd03228	18
cd02933	16
cd00145	14
cd01653	10
