# Supplementary material for: Identifying and Seeing beyond Multiple Sequence Alignment Errors Using Intra-Molecular Protein Covariation
Source: PLoS One. 2010 Jun 28;5(6):e11082. doi: 10.1371/journal.pone.0011082 (PMC2893159; doi:10.1371/journal.pone.0011082)
Supplement: Table S2 — Sequences removed from cd00300 because of poor alignment to structure alignment. Not all possibly erroneous sequences were removed in order to meet the cutoff for minimum number of sequences for covariance methods. (0.08 MB PDF) [file pone.0011082.s005.pdf]

gi	annotation	reason for removal
987693	lactate dehydrogenase	have to force global alignment
15899314	lactate dehydrogenase	have to force global alignment
16803097	hypothetical (mdh/ldh like)	does not match ungapped segments
17367527	probable malate dehydrogenase	paralogous sequence
17367610	probable malate dehydrogenase	paralogous sequence
29826879	lactate dehydrogenase	partial sequence
33860171	lactate dehydrogenase	have to force global alignment
38233041	putative lactate dehydrogenase	have to force global alignment
47217234	unnamed protein product	no match at N terminus
54020211	hypothetical protein (mdh/ldh- like)	have to force global alignment
58337781	lactate dehydrogenase	have to force global alignment
70606086	lactate/malate dehydrogenase	have to force global alignment
92089264	has been removed from database	have to force global alignment
