# Supplementary material for: Identifying and Seeing beyond Multiple Sequence Alignment Errors Using Intra-Molecular Protein Covariation
Source: PLoS One. 2010 Jun 28;5(6):e11082. doi: 10.1371/journal.pone.0011082 (PMC2893159; doi:10.1371/journal.pone.0011082)
Supplement: Table S3 — Scoring cutoffs for arbitrary accuracy in CDD alignments. CDD alignments were examined and those that contained ≥150 sequences, ≥50 nongapped positions and where each method made at least one true prediction were identified at an accuracy of 80% or higher. There were 100 such protein families and they are identified in Table S4. The cutoff values for each measure at which at least the fraction of pairs identified were in contact is given along with the mean number of pairs identified in each protein family. Previous work had identified a cutoff of 4.5 for MIp, and the analysis here suggests that cutoff is appropriate for ∼80% accuracy. Only pairs 10 or more positions apart in sequence are included to prevent proximity in sequence from biasing results. (0.02 MB PDF) [file pone.0011082.s006.pdf]

	50%	$\overline{N}_{50\%}$	60%	$\overline{N}_{60\%}$	70%	$\overline{N}_{70\%}$	80%	$\overline{N}_{80\%}$	90%	$\overline{N}_{90\%}$
ΔZp	1.28	18.47	1.73	13.75	2.43	10.39	2.91	7.79	3.58	5.46
Zp	3.07	17.44	3.59	11.98	4.12	8.76	4.63	6.64	5.14	4.42
Zpx	2.59	22.05	2.89	16.48	3.26	12.5	3.63	9.06	3.94	6.57
