# Supplementary material for: Identifying and Seeing beyond Multiple Sequence Alignment Errors Using Intra-Molecular Protein Covariation
Source: PLoS One. 2010 Jun 28;5(6):e11082. doi: 10.1371/journal.pone.0011082 (PMC2893159; doi:10.1371/journal.pone.0011082)
Supplement: Table S4 — Venn Diagram Alignments. Alignments curated according to Materials and Methods, used to generate Figure 7 and Table S3. (0.09 MB PDF) [file pone.0011082.s007.pdf]

cd00338	pfam00239	cd00311	cd00105
cd00198	cd02039	pfam00092	cd04496
pfam00027	cd00158	pfam00665	cd00882
cd00291	cd05380	pfam00104	cd00096
cd02961	cd04100	cd01876	pfam01627
COG0589	cd00086	cd00082	cd00063
cd00208	cd00037	pfam04055	pfam07714
cd04371	pfam02826	cd00043	cd00751
cd00352	cd04724	pfam03466	cd00668
cd00446	pfam00004	cd03442	cd04730
cd02980	cd00293	cd00382	pfam02210
cd03586	cd00591	cd00636	cd00154
cd00570	smart00034	cd02966	cd00204
cd02801	pfam02502	cd00254	cd00448
cd01182	pfam00570	cd00176	COG0451
cd00403	smart00091	cd00030	cd03441
cd00173	COG1609	cd00992	pfam05193
cd00031	pfam00307	cd00090	smart00385
cd02869	cd00919	pfam00753	cd00299
cd02042	cd00159	cd01911	cd00130
cd00433	pfam00227	pfam00496	pfam00149
cd00405	cd00041	pfam00582	COG1024
pfam09346	cd01898	cd01342	pfam01636
cd00084	cd00712	cd01610	COG0778
cd00383	COG0436	cd00564	cd00093
